# Asssociation between occupational stress, work arrangements, and depression in men: Review of observational studies 2020–2025

**DOI:** 10.1097/MD.0000000000050735

**Published:** 2026-09-18

**Authors:** Haewon Byeon

**Affiliations:** a Worker’s Care & Digital Health Lab, Department of Future Technology, Korea University of Technology and Education, Cheonan, South Korea.

**Keywords:** depressive symptoms, long working hours, men, occupational stress, shift work

## Abstract

**Background:**

Men may be particularly vulnerable to cumulative occupational stress because of breadwinner expectations, employment in high-risk industries, night work, and delayed help-seeking. However, evidence linking occupational stress and nonstandard work arrangements to depression in men remains inconsistent because of heterogeneous study designs, exposure definitions, and outcome measures.

**Methods:**

This review synthesized observational studies published between 2020 and 2025 that examined the effects of psychosocial occupational stress, shift or night work, and long working hours on clinically diagnosed depression or depressive symptoms in men. Studies were eligible if they included male-only cohorts or reported sex-stratified results. PubMed, Scopus, Web of Science, Korean Citation Index, and Research Information Sharing Service, were searched for records published from January 1, 2020, through February 2, 2026. Risk of bias was assessed using Risk Of Bias In Non-randomized Studies: of Interventions.

**Results:**

Eleven observational studies were included. Overall risk of bias was rated Moderate in 36.4% (4/11) and Serious in 63.6% (7/11) of the studies; none were rated Critical. High job strain was associated with an increased risk of major depressive episodes in men. Shift work was associated with higher depression risk in the UK Biobank, whereas a German cohort showed a positive but uncertain association. In Korean studies, associations between night or shift work and depression generally weakened after adjustment for confounders. In contrast, psychosocial stressors: including high job demands, interpersonal conflict, job insecurity, and rigid workplace culture: remained strongly associated with incident depression. Long working hours also significantly increased depression risk among young adults.

**Conclusion:**

These findings indicate that depression risk in men reflects interactions between work schedules and qualitative occupational stressors rather than work arrangements alone. Future studies should employ longitudinal designs, standardized depression assessments, precise and repeated exposure measurements, and rigorous confounder control. Male-specific analyses stratified by age, occupation, and employment stability are also needed to strengthen causal inference.

## 1. Introduction

Employment consumes the majority of time and energy in the daily lives of adult men. Consequently, the occupational stress experienced during this process: such as job demand–control imbalances, organizational dysfunction, and job insecurity: along with nonstandard work arrangements (shift work, night work, long hours), have been repeatedly identified as critical social and environmental determinants of depression.^[1–5]^ Men are particularly prone to accumulating stress within structural and cultural contexts defined by breadwinner expectations, gender role norms, competitive pressure, and restricted emotional expression or help-seeking behaviors.^[6]^ As a result, even when depression occurs, medical intervention is frequently delayed. Therefore, determining how variations in stress and work schedules alter depression risk is essential for designing workplace-based preventive strategies and policies that extend beyond individual-level treatment.

The link between occupational stress and depression is supported by robust theoretical frameworks. Karasek’s Job Demand–Control (job strain) model posits that the combination of high psychological demands and low decision latitude precipitates psychological burden, potentially leading to emotional exhaustion and depression.^[7,8]^ Similarly, the Korean Occupational Stress Scale (KOSS), widely used in Korean corporate environments, captures granular organizational factors including job insecurity, organizational systems, workplace culture, and interpersonal conflict.^[9,10]^ Empirical evidence reinforces these models; a cohort study reported that high job strain more than doubled the subsequent risk of major depressive episodes (MDE) in men.^[11]^ This suggests that male depression risk is sensitive not only to the absolute level of stress but also to its trajectory and the erosion of employment stability over time.

Work arrangements and working hours constitute another critical axis influencing depression. Beyond simple workload increases, these factors may promote depression through mechanisms such as circadian rhythm disruption, poor sleep quality, social isolation, and increased family or interpersonal conflict.^[12–20]^ Large-scale cohort data indicate that shift work is associated with an elevated risk of depression compared to non-shift work,^[12]^ and Korean national survey data show an increased odds ratio (OR) for high-risk depression (assessed via 9-item Patient Health Questionnaire [PHQ-9]) among men engaged in night or shift work.^[19]^ However, the relationship between shift work and depression is heterogeneous, varying by industry, occupation, shift pattern (e.g., fixed night vs rotating), individual adaptability (chronotype), and organizational protective factors. Indeed, some cohort studies report results with high uncertainty,^[13]^ suggesting that the effects of work arrangements require interpretation that goes beyond binary comparisons and accounts for measurement context.

The association between long working hours and depression is also frequently cited, yet the evidence for men is inconsistent. Cross-sectional analyses of young workers have demonstrated a clear dose-response relationship, where longer hours significantly increase the odds of depression.^[16]^ Conversely, long-term panel studies have reported that while excessive work hours increase depression risk in the general population, the effect estimate for men specifically is often negligible.^[15]^ Furthermore, research employing instrumental variable (IV) approaches to account for endogeneity: such as the tendency for depressed individuals to reduce hours or exit the labor market: has tested the nonlinearity of this relationship.^[18]^ These mixed findings indicate that observed associations depend heavily on age, occupational group, job security, job demands, and the specific depression instrument used (e.g., diagnostic interview vs screening tool vs single-item).

A significant limitation of the current literature is the lack of “direct” male-specific evidence. While some studies strictly track incident depression in male cohorts,^[21]^ others suffer from low male representation,^[20]^ provide limited sex-stratified data, or treat depression merely as a mediator or covariate rather than a primary outcome.^[14,17]^ Additionally, the operationalization of depression varies widely, ranging from Composite International Diagnostic Interview-Short Form (CIDI-SF)-based MDEs and medical records to screening tools (Patient Health Questionnaire, Center for Epidemiologic Studies Depression Scale [CES-D]) and single-item assessments.^[11,12,16,21]^ This heterogeneity conflates clinically distinct outcomes under the single term “depression.” Therefore, a systematic review is needed to synthesize these findings, critically evaluating not just the direction of association but also the definitions of exposure and outcome, the temporal sequence of study designs, and the specificity of the analysis to men.

This study systematically reviews observational research published between 2020 and 2025 to determine the association between occupational stress, work arrangements (shift/night work, long hours), and depression in men.^[11–21]^ We aimed to clarify the scope and limitations of current evidence by synthesizing data on: study design and male representativeness; the measurement of exposures and control group definitions; the heterogeneity of depression outcomes and effect estimates; and risk of bias using standardized assessment tools. Our ultimate goal is to identify high-risk exposure profiles: such as deteriorating job security, intensifying demands, or specific adverse work schedules: to provide a foundational basis for targeted mental health policies and preventive interventions.

## 2. Material and methods

### 2.1. Study design and analysis strategy

We conducted this study to investigate the impact of occupational stress and precarious work arrangements (e.g., shift work, night labor, long working hours) on depression and depressive symptoms in male workers. We restricted our analysis to observational studies published between January 1, 2020, and December 31, 2025. This timeframe was selected to capture the most recent data reflecting the rapidly shifting labor landscape surrounding the COVID-19 pandemic.

To ensure transparency, we adhered to the Preferred Reporting Items for Systematic Reviews and Meta-Analyses (PRISMA) guidelines throughout the review process. However, given the substantial heterogeneity in exposure variables and outcome measures across the collected literature, we determined that a qualitative synthesis was more appropriate than a quantitative meta-analysis. The final synthesis incorporates data from cohort, panel, and cross-sectional study designs. This review was not prospectively registered in International Prospective Register of Systematic Reviews. An internal working protocol specifying the eligibility criteria, search concepts, extraction fields, and Risk Of Bias In Non-randomized Studies: of Interventions (ROBINS-I) domains was prepared before full-text assessment; however, registration was considered only after screening had begun. Because retrospective registration would not provide the bias-reduction benefit of a prospective record, we did not register the review after completion. This omission is reported transparently.

### 2.2. Eligibility criteria

Our review encompassed adult workers across all industries and occupations. Since our primary objective was to isolate the mental health characteristics of men, we selected only those studies that analyzed exclusively male cohorts or provided gender-stratified analyses allowing for the independent extraction of male data.

We approached exposures through 2 primary dimensions.

A. Occupational Stress: Psychosocial factors including job strain, job demand–control imbalances, and job insecurity.
B. Work Arrangements: Physical factors such as shift work, night work, and nonstandard work schedules.
C. Comparators (Control Groups) were generally defined as fixed day workers or those adhering to standard working hours (e.g., 35–40 hours per week), depending on the definitions used in individual studies.
D. Primary Outcomes included clinically diagnosed MDE as well as depressive symptoms assessed via validated screening tools such as the PHQ-9 or CES-D. We did not exclude studies relying on self-reported questionnaires (including single-item measures), as capturing subjective depressive states prior to clinical diagnosis remains highly relevant to occupational health surveillance.
E. Exclusion Criteria: To maintain clarity in causal inference, we excluded interventional studies (e.g., randomized controlled trials) and simple review articles. Furthermore, we excluded studies where independent data extraction for men was impossible or where bibliographic information was unclear.

### 2.3. Search strategy

We performed a comprehensive search of domestic and international databases through February 2, 2026. This cutoff was strategic, ensuring the complete capture of literature published through the end of 2025. We queried PubMed, Scopus, and Web of Science for English literature, and Korean Citation Index, Research Information Sharing Service, and DBpia for Korean literature.

The search strategy combined terms from 4 conceptual blocks using Boolean operators.

A. Occupational Stress: occupational stress OR job strain OR work stress OR job demand OR effort-reward imbalance.
B. Work Arrangements/Hours: shift work OR night shift OR long working hours OR working-time OR work schedule OR nonstandard work.
C. Depression: depression OR depressive symptoms OR major depressive disorder OR PHQ-9.
D. Gender (Male): male OR men OR gender OR sex difference.

To address the potential limitations of electronic database indexing, we supplemented our search by manually cross-referencing the bibliographies of primarily selected papers (citation chasing) to identify additional relevant studies.

### 2.4. Study selection

After deduplication, 2 independent reviewers screened all titles and abstracts and independently assessed potentially eligible full texts against the prespecified criteria. Each reviewer recorded the reason for full-text exclusion on the structured form. Disagreements were resolved through discussion; any unresolved case was adjudicated by the author. Final decisions were documented in the PRISMA flow diagram. The reviewers’ affiliations and nonauthor roles are reported in the Acknowledgements.

## 3. Data extraction and quality assessment

### 3.1. Data extraction protocol

To minimize inter-rater variability and ensure data consistency, we extracted information using a predefined, structured form. We categorized the extracted data into general study characteristics and analytical metrics. First, we recorded bibliographic details (author, publication year), the country of origin, and the data source. We then scrutinized the study design and demographic characteristics of the sample, including total number of participants, proportion of men, and mean age.

Given that our primary objective was to clarify the link between exposure and outcome, we rigorously extracted the operational definitions of “occupational stress” and “work arrangements” used in each study, along with the criteria for their respective reference groups. We also identified the specific measurement tools and clinical cutoff values used to define depression. For the final effect sizes, we prioritized OR, Relative Risks (RR), or regression coefficients specific to male cohorts. This focus allowed us to isolate male-specific occupational health characteristics, independent of the biological or social confounding factors often present in mixed-gender analyses.

### 3.2. Risk of bias assessment

Because the selected literature consisted entirely of observational studies without experimental intervention, we employed the ROBINS-I tool to evaluate potential threats to internal validity.

We assessed bias across 7 distinct domains covering the entire research process, A. Confounding, B. Selection of participants, C. Classification of interventions (exposures), D. Deviations from intended interventions, E. Missing data, F. Measurement of outcomes, G. Selection of the reported result.

We graded each domain on a 5-point scale ranging from “Low” to “Critical” risk, or “No information.” For the overall risk of bias, we adopted a conservative approach: if any single domain was rated as high-risk, the overall quality of the study was downgraded to that level. This rigorous verification procedure is essential to prevent the overestimation of positive associations that can occur during qualitative synthesis.

## 4. Results

### 4.1. Study characteristics and male representation

**Figure 1.**
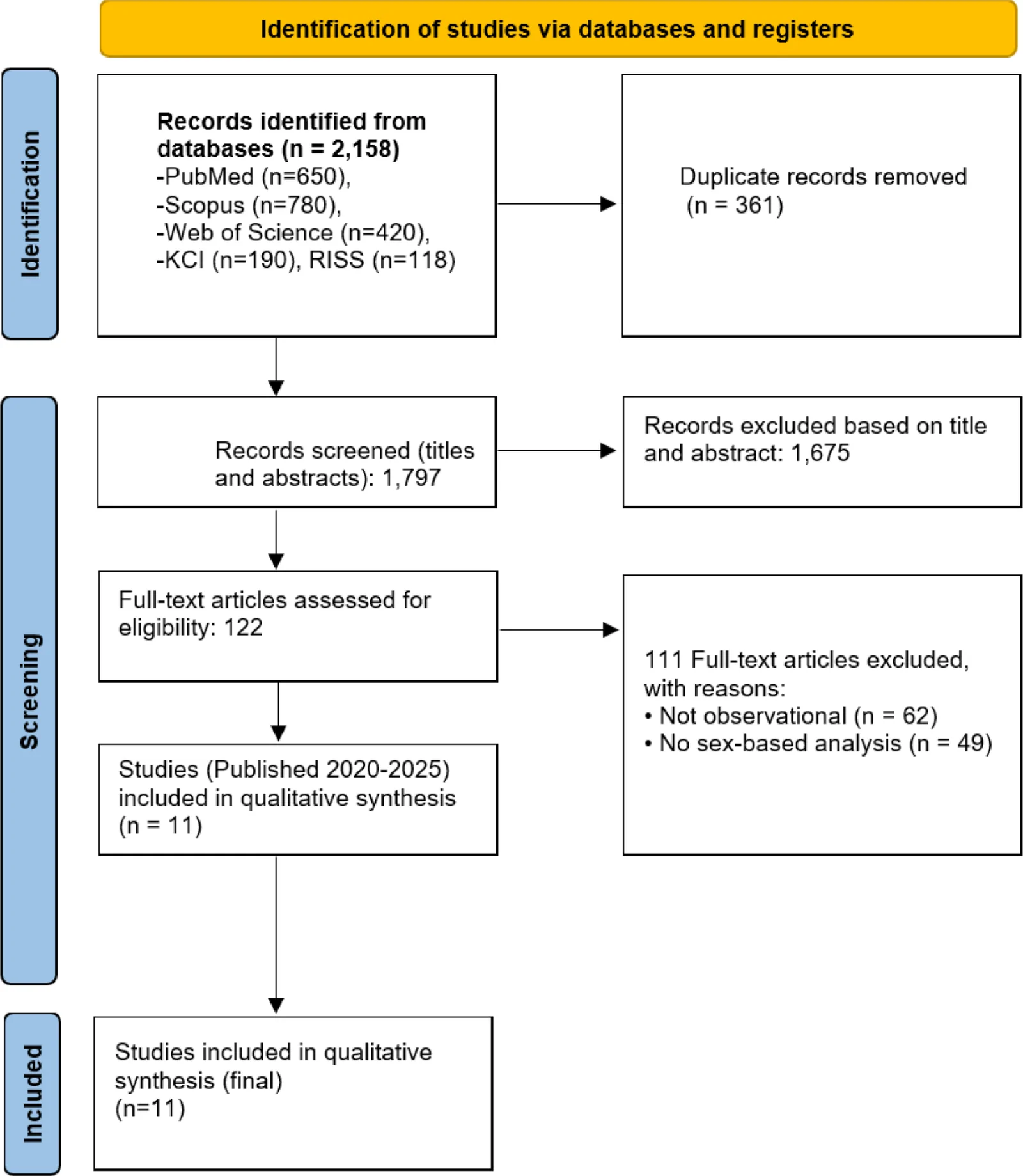
PRISMA flow diagram showing study identification, duplicate removal, screening, full-text eligibility assessment, exclusions, and the final inclusion of 11 observational studies.

The PRISMA flow diagram is presented in Figure 1. We identified 11 observational studies comprising large population-based cohorts, workplace-based panels, and national cross-sectional surveys (Table 1). Sample sizes varied widely, ranging from 405 participants in a repeated-measures occupational panel to 175,543 in a general population cohort, with 1 long-running national panel contributing over 18,000 person-observations. Male representation fluctuated significantly across studies, from a low of 31.9% in a semiconductor industry panel to 88.7% in a physician survey, although several datasets approached gender balance. Crucially, the specificity of evidence regarding men varied. One cohort recruited men exclusively: specifically manufacturing workers free of baseline depression: allowing for direct inference on stress trajectories.^[21]^ In contrast, other large datasets either provided mixed-sex estimates without formal sex-stratification or reported male-stratified findings only for selected exposure contrasts.^[12,13,15,17,18]^ This inconsistency hinders the direct comparability of male-specific effect estimates across the full evidence set.

**Table 1:** Characteristics of the analyzed studies.

Ref.	Study (Author, Yr)	Country/data source	Design	Sample (N/Male %/ Age)	Exposure (Occupational stress/ work arrangements)	Comparator (Reference)	Depression outcome (Tool/ cutoff)	Key results
^[11]^	Matthews et al 2021	USA/ MIDUS	Cohort (~9-year follow-up)	N = 1581 (Male: 805, 50.9%); Mostly mid-life (peak 46–55 yr)	Job strain: Karasek model (Demands + Control). Family strain: 4 items (e.g., “Family lets me down”)	Low job strain/ low family strain	MDE: WHO CIDI-SF (12-month MDE at follow-up)	Men: High job strain associated with increased MDE risk (RR = 2.14, 95% CI 1.14–4.03). Family strain was not significant (RR = 1.12, 0.55–2.27).
^[12]^	Xu et al 2023	UK/ UK Biobank (Recruited 2006–2010)	Cohort (Prospective)	N = 175,543 (Male: 88,290, 50.3%); Mean age 52.6 ± 7.1	Shift work: type, frequency, duration (including night work)	Non-shift work	Depression/Anxiety: incident cases via medical records (ICD codes)	Shift workers showed increased risk of depression vs non-shift workers (Fully adjusted HR = 1.22, 95% CI 1.18–1.26). Risk also increased for comorbidity (Depression + Anxiety, HR = 1.16).
^[13]^	Behrens et al 2021	Germany/ Heinz Nixdorf Recall	Cohort (5 & 10 yr)	5-yr sample: Male 896, Female 340; Male mean age 56–58 (Ever-shift group: 56.8 ± 4.1)	Shift work history: ever vs Never/ <1 yr (includes night work)	Never/short duration (<1 yr)	Depressive symptoms: CES-D based incident cases (excluding baseline) + PHQ auxiliary	Men: Risk for “ Ever shift work” showed an upward trend but with uncertainty: 5-yr RR = 1.43 (0.82–2.49); 10-yr RR = 1.39 (0.61–3.17).
^[14]^	Park et al 2022	Korea/ emergency physicians	Cross-sectional (survey)	N = 954 (Male: 846, 88.7%); Mean age 42.5 ± 6.1	Monthly night shift days; family function (APGAR); sleep quality (PSQI)	Fewer night shift days (continuous model)	Depression: PHQ-9 (cutoff ≥ 11 used in study)	While the primary outcome was Excessive daytime sleepiness (EDS), prevalence of depression was 28.4%. night shift frequency + depression were associated with EDS risk.
^[15]^	Baek et al 2025	Korea/ National Panel (2006–2022)	Longitudinal (Repeated-measures)	N = 4941 (18,531 observations); Male 60.1%; Middle-aged to older adults (45+)	Weekly working hours: 35–40, 41–48, 49–54, ≥55	35–40 h	Depressive symptoms: CES-D-10 (Cutoff ≥ 10)	Overall: ≥55h associated with depression (RR = 1.12, 1.01–1.24). Men: association was not significant (RR = 1.02, 0.89–1.16); effect was stronger in women.
^[16]^	Park et al 2020	Korea/ youth panel 2007 (2012 follow-up)	Cross-sectional (at follow-up)	N = 3332 (Male: 1629, 48.9%); Age 20–35	Weekly working h: 41–50, 51–60, >60	31–40 h	Depression: single-item (depressed mood/hopelessness ≥ 2 wk)	Dose-response relationship: 41–50h (OR = 1.97); 51–60h (OR = 2.56); >60h (OR = 4.20) compared to standard h.
^[17]^	Kim et al 2022	Korea/ KNHANES 2007–2018	Cross-sectional	N = 33,047 (Male ~52.6%); Mean age ~47–49	Shift patterns (Fixed evening/night, rotating), working hours, sleep duration	Day work	Depressive mood: single-item (Self-reported ≥ 2 wk)	Established a pathway where shift work influences suicidal ideation mediated by depressive mood (measured via single-item).
^[18]^	Niu et al 2024	China/ National Survey	Cross-sectional	Observations ~11,655–12,452	Weekly working hours: continuous + squared term (IV: exogenous impact of automation)	Optimal working hours (Estimated)	Mental health: self-rated depression severity (ordinal scale)	Confirmed a U-shaped relationship between working hours and depression severity.
^[19]^	Lee et al 2021	Korea/ KNHANES 2014/16/18	Cross-sectional	N = 10,851 (Male: 5548, ~51.1%); All age groups	Work Type: night/shift work (includes evening, 24h, irregular, night 9pm–8am)	Day work	Depression Risk: PHQ-9 (High-risk group)	Men: night/shift work adjusted OR = 1.407 (0.937–2.113); unadjusted OR = 1.549 (1.038–2.311). Subjective stress perception Adjusted OR = 0.932 (0.785–1.107).
^[20]^	Kim et al 2020	Korea/ semiconductor Panel	Cohort (Repeated-measures)	405 completed 3 waves (Male: 129, 31.9%); Mean age 30.8 ± 0.3 (in 2009)	Occupational stress: KOSS-SF (24 items) subdomains (Demand, control, conflict, insecurity, etc)	Relatively low/ stable level	Depressive symptoms: CES-D (continuous score)	Male fixed-effects: only job insecurity was significantly associated with worsening symptoms (B = 0.048, *P* = .004). (Cross-sectional analysis showed Job Demand B = 2.26, *P* = .026).
^[21]^	Kim et al 2023	Korea/ manufacturing Cohort (2018–2019)	Cohort (incident depression)	Men N = 1837 (Free of baseline depression); 98 incident cases (5.3%); Age 20–59	Stress change (KOSS-SF): Increase vs same; transitions (Low to low, low to high, high to low, high to high)	Stress ‘ same’ or ‘ low to low’	Incident depression: PHQ-2	Men (Increase vs Same): Job Demand (OR 2.46), interpersonal conflict (OR 2.16), organizational system (OR 1.83), Workplace culture (OR 2.42). Men (Low to High): Job Demand (OR 4.34), job insecurity (OR 3.21). Men (high to high): job demand (OR 5.86).

CES-D = Center for Epidemiologic Studies Depression Scale, CI = confidence interval, HR = hazard ratio, IV = instrumental variable, KOSS = Korean Occupational Stress Scale, MDE = major depressive episode, OR = odds ratio, PHQ = Patient Health Questionnaire, RR = relative risk.

### 4.2. Operationalization of exposures: stress versus schedule

Operational definitions of exposures fell into 2 primary domains: psychosocial job stress constructs and metrics of work schedule or duration.^[11,20,21]^ Psychosocial stress was predominantly measured using established theoretical frameworks or validated scales, such as Karasek’s Job Demand–Control model in a U.S. cohort^[11]^ and the KOSS in Korean workplace studies.^[20,21]^ The KOSS captures granular subdomains including job demand, interpersonal conflict, organizational systems, and workplace culture. Conversely, work schedule exposures focused on shift or night work patterns (e.g., fixed vs rotating) and weekly working hours.^[12–19]^ Working hours were modeled categorically, continuously, or via nonlinear approaches, with 1 study employing an IV strategy to address endogeneity. While reference groups generally represented low-exposure conditions, such as day work or standard hours, the granularity of measurement varied from binary classifications to multidimensional longitudinal typologies (e.g., stable low vs increasing stress).^[12,15,19,21]^ This methodological divergence drives between-study variation, as schedule metrics primarily capture circadian disruption and dosage, whereas psychosocial scales isolate proximal determinants of symptoms such as demand–control imbalances and organizational climate.^[11,20,21]^

### 4.3. Heterogeneity in depression outcomes

The assessment of depression involved substantial methodological heterogeneity, precluding strict comparability of case definitions and effect sizes (Table 1). Diagnostic endpoints included MDE derived from the World Health Organization CIDI-SF and diagnoses extracted from electronic medical records.^[11,12]^ Other studies utilized validated screening scales with study-specific thresholds, such as the PHQ-9, 10-item CES-D, or 2-item Patient Health Questionnaire, while 1 panel modeled CES-D scores continuously.^[14,15,20,21]^ A subset of studies relied on coarser measures, including single-item indicators (e.g., depressed mood $\ge$ 2 weeks) or ordinal self-rated severity scales.^[16–18]^ These variations increase the risk of outcome misclassification and complicate the synthesis of findings with those derived from standardized instruments. Consequently, the evidence base spans a wide spectrum from clinical diagnoses to subjective self-reports, necessitating cautious interpretation of pooled findings given the lack of a uniform clinical anchor.

### 4.4. Core findings with emphasis on male-specific inference

Across the reviewed studies, the general direction of association suggested that higher occupational stress and adverse working-time patterns increase depression risk. However, the strength and precision of these estimates varied significantly depending on exposure definitions, outcome measures, and the extent of male representation. regarding psychosocial stress, a U.S. cohort study demonstrated that high job strain substantially increased the risk of MDE among men (RR = 2.14; 95% confidence interval [CI] 1.14–4.03), whereas family strain showed no statistically significant association in this group.^[11]^ In Korean workplace analyses, male-specific signals were most consistent when modeling stress trajectories over time. Among male manufacturing workers initially free of depression, increasing stress: specifically regarding job demands, interpersonal conflict, organizational systems, and workplace culture: was associated with higher odds of incident depression. The risk was particularly pronounced for those transitioning from low to high stress or maintaining consistently high stress levels in key subdomains.^[21]^ Similarly, in a repeated-measures panel of semiconductor workers, male fixed-effects estimates identified job insecurity as the stressor most robustly linked to worsening depressive symptoms ($B = 0.048$, $*P* = .004$), indicating that within-person deterioration in perceived security is a potent depressogenic factor for men.^[20]^

Evidence linking work schedules and hours to depression in men was less consistent. Large-scale cohort data indicated an overall association between shift work and elevated depression risk (fully adjusted hazard ratio = 1.22; 95% CI 1.18–1.26); however, this summary effect was not stratified by sex in the extracted data.^[12]^ In contrast, a German cohort consisting predominantly of men yielded point estimates above 1.0 for the association between shift work history and depression risk at 5- and 10-year follow-ups, but the wide CIs precluded definitive conclusions regarding the magnitude of male-specific risk.^[13]^ Findings on working hours were similarly mixed. A long-term Korean panel suggested a modest overall increase in depression risk for those working $\ge$55 hours/week, yet the male-stratified estimate was near-null (RR = 1.02; 95% CI 0.89–1.16), implying potential sex differences or differential confounding by employment regime.^[15]^ Conversely, cross-sectional analyses of younger Korean workers showed a strong dose-response relationship, where working >60 hours/week significantly increased the odds of depressed mood compared to standard hours (OR $\approx$ 4.20).^[16]^ However, the reliance on a single-item outcome and the lack of temporal precedence in the latter study limits causal inference. Further variability was observed in studies employing IV approaches (suggesting a U-shaped relationship) or mediation frameworks, underscoring that differences in analytic strategy affect the direct applicability of these estimates to male populations.^[17,18]^

### 4.5. Implications for synthesis focused on men

From a synthesis perspective, the most policy-relevant and methodologically informative evidence is derived from studies that provide explicit male-stratified estimates or utilize male-only cohorts with incident depression endpoints.^[11,15,19–21]^ Even within this subset, inference is constrained by heterogeneity in depression measurement (ranging from diagnostic episodes to single-item screens), exposure granularity (binary vs multidomain scales), and study design. These variations prevent the pooling of effect sizes into a single “male occupational stress $\to$ depression” parameter. Nevertheless, a consistent theme emerges from the higher-quality studies: worsening psychosocial stress: particularly in domains related to job demands, insecurity, and organizational climate: demonstrates clearer and often stronger associations with depression onset than static categorizations of work schedules alone. This suggests that male mental health risk may be less dependent on the physical arrangement of work hours and more sensitive to the dynamic deterioration of perceived control, stability, and workplace culture.^[11,20,21]^

### 4.6. Risk of bias assessment using ROBINS-I

We assessed the risk of bias for the 11 included observational studies^[11–21]^ using the ROBINS-I tool. In terms of Overall Bias, 36.4% (4/11) of the studies were classified as Moderate,^[11,12,15,21]^ while 63.6% (7/11) were classified as Serious.^[13,14,16–20]^ No studies were deemed to have a Critical risk of bias. Studies rated as Moderate typically employed longitudinal designs that established temporal precedence or utilized advanced analytic techniques (e.g., panel analysis, multiple imputation) to partially mitigate key biases.^[11,12,15,21]^ Conversely, studies rated as Serious often suffered from limitations inherent to cross-sectional designs, such as potential reverse causality, inadequate control for confounding, and cumulative selection bias due to attrition or exclusion criteria, all of which weaken the strength of causal inference.^[13,14,16–20]^

### 4.7. Bias due to confounding

The risk of bias due to confounding was rated as Moderate in 63.6% (7/11)^[11,12,15,17,18,20,21]^ and Serious in 36.4% (4/11)^[13,14,16,19]^ of the studies. Serious ratings were primarily assigned when the set of adjustment variables was limited,^[16,19]^ cross-sectional designs failed to control for sufficient occupational, health, and socioeconomic factors amidst unclear temporal sequences,^[14]^ or long-term follow-up studies remained susceptible to significant residual confounding.^[13]^ Moderate ratings were assigned to studies that attempted to control for time-invariant confounders through extensive covariate adjustment (sociodemographic, lifestyle, health factors) or fixed-effects panel analysis.^[11,12,15,17,18,20,21]^ However, even in these cases, it was difficult to completely rule out residual confounding from unmeasured factors such as specific job characteristics, mental health history, sleep quality, and the physical work environment.^[11,12,15,20,21]^

### 4.8. Bias in selection of participants

Selection bias was rated as Moderate in 72.7% (8/11)^[11,12,14–18,21]^ and Serious in 27.3% (3/11).^[13,19,20]^ Serious bias arose when the analysis sample was substantially reduced due to the mass exclusion of nonresponders or ineligible participants,^[19]^ or when high attrition rates resulted in analyses restricted to complete cases, where drop-out might be correlated with both exposure and outcome.^[20]^ Furthermore, the cumulative “healthy worker effect”: driven by retirement, death, or nonparticipation in long-term cohorts: also contributed to selection bias.^[13]^ Even studies rated as Moderate faced potential threats to external validity and selection bias due to the representativeness of volunteer-based cohorts or the selectivity inherent in single-workplace or single-occupation samples.^[12,21]^

### 4.9. Bias in classification of exposures

We rated the bias in exposure classification (occupational stress and work arrangements) as Moderate for all 11 studies.^[11–21]^ Most studies relied on self-reported questionnaires (e.g., KOSS, work schedule items) to measure work type (night/shift vs day) or job stress, introducing a risk of misclassification.^[11,12,15,19–21]^ Specifically, the lack of detail regarding shift patterns (irregularity, frequency, duration) or the dichotomization of continuous stress scores based on medians may lead to misclassification around the cut-points.^[12,21]^ However, we did not classify this as “Serious” because such misclassification is likely non-differential, tending to bias effect estimates toward the null (conservative bias).^[19,21]^

### 4.10. Bias due to deviations from intended exposures

Given the observational nature of these studies, there is no “intended intervention” as in randomized trials; thus, this domain was generally rated as Low to Moderate. However, bias may arise if work arrangements or job stress change during the follow-up period but are treated as fixed based on a single baseline measurement (time-varying exposure).^[11–13]^ Studies that explicitly modeled changes in exposure (e.g., stress typologies: increasing, low$\to$high, high$\to$high) were considered to have partially mitigated this risk.^[21]^

### 4.11. Bias Due to Missing Data

Bias due to missing data was rated as Low/Low-Moderate in 9.1% (1/11),^[15]^ Moderate in 63.6% (7/11),^[11,12,14,16–18,21]^ and Serious in 27.3% (3/11).^[13,19,20]^ Serious ratings were assigned when substantial exclusions occurred during participant selection^[19]^ or when significant attrition in long-term follow-up led to complete-case analysis.^[20]^ Cumulative censoring and attrition in long-term cohorts can distort estimates if retention probabilities depend on exposure and mental health status.^[13]^ In contrast, studies that explicitly applied missing data strategies, such as Multiple Imputation by Chained Equations, were rated as having lower risk.^[15]^

### 4.12. Bias in Measurement of Outcomes

Bias in outcome measurement was distributed as Low-Moderate in 18.2% (2/11),^[11,14]^ Moderate in 54.5% (6/11),^[12,13,15,19–21]^ and Serious in 27.3% (3/11).^[16–18]^ Serious bias was identified in studies measuring depression via single items (yes/no or single Likert scale), which pose a high-risk of misclassification due to poor validity and sensitivity.^[16–18]^ Moderate ratings were given to studies using widely accepted screening tools (e.g., 2-item Patient Health Questionnaire/9, CES-D) that rely on self-reporting, leaving the possibility of reporting bias.^[12,13,15,19–21]^ Studies utilizing diagnostic interviews (CIDI-SF) or standardized screening tools with clear cutoff values were rated as having relatively lower bias.^[11,14]^

### 4.13. Bias in Selection of the Reported Result

The risk of selective reporting(table 2) was rated as Moderate in 81.8% (9/11),^[11–13,15–20]^ No Information in 9.1% (1/11),^[14]^ and Low-Moderate in 9.1% (1/11).^[21]^ The prevalence of Moderate ratings reflects the high analytical flexibility in many studies: involving multiple exposures (subdomains/work types), various models (stepwise adjustment), and subgroup/mediation analyses: making full comparison with prespecified analysis plans difficult.^[11–13,15–20]^ One study was categorized as “No Information” because depression was treated primarily as an explanatory variable for another outcome rather than a primary endpoint, complicating the assessment of reporting bias.^[14]^ A study that systematically presented incident depression alongside stress changes in tables and models was rated as having lower risk.^[21]^

**Table 2:** Risk of bias assessment results using ROBINS-I.

Ref.	Confounding	Selection	Classification	Deviations	Missing data	Outcome measurement	Selective reporting	Overall risk
^[11]^	Moderate	Moderate	Moderate	Moderate	Moderate	Low to Moderate	Moderate	Moderate
^[12]^	Moderate	Moderate	Moderate	Moderate to Serious	Moderate	Moderate	Moderate	Moderate
^[13]^	Serious	Serious	Moderate	Moderate	Serious	Moderate	Moderate	Serious
^[14]^	Serious	Moderate	Moderate	Moderate	Moderate	Low to moderate	No information	Serious
^[15]^	Moderate	Moderate	Moderate	Low to Moderate	Low to Moderate	Moderate	Moderate	Moderate
^[16]^	Serious	Moderate	Moderate	Moderate	Moderate	Serious	Moderate	Serious
^[17]^	Moderate	Moderate	Moderate	Moderate	Moderate	Serious	Moderate	Serious
^[18]^	Moderate	Moderate	Moderate	Moderate	Moderate	Serious	Moderate	Serious
^[19]^	Serious	Serious	Moderate	Low	Serious	Moderate	Moderate	Serious
^[20]^	Moderate	Serious	Moderate	Low	Serious	Moderate	Moderate	Serious
^[21]^	Moderate	Moderate	Moderate	Low	Moderate	Moderate	Low to moderate	Moderate

### 4.14. Overall interpretation

In summary, while current evidence suggests that increased occupational stress and non-day (night/shift) work are associated with depression,^[11–13,15,19,21]^ the causal interpretation and generalizability of these effect estimates are limited by unresolved confounding and selection biases in many studies.^[13,19,20]^ Regarding male-specific inference, relatively strong associations were observed in a cohort tracking men exclusively.^[21]^ However, because some studies had low male representation^[20]^ or relied on cross-sectional designs where the stability of gender-stratified results is uncertain,^[19]^ additional high-quality longitudinal research is required to precisely determine the “magnitude and mechanisms” of these associations in men.^[11,12,15,19–21]^

Risk of bias categories are based on ROBINS-I guidelines: Low, Moderate, Serious, Critical, and No information. “Deviations” refers to bias due to deviations from intended exposures.

## 5. Discussion

This systematic review synthesized 11 observational studies published between 2020 and 2025. While the aggregate evidence supports a general association between occupational stress, nonstandard work arrangements (shift work, night shifts, long hours), and depression in men, the magnitude and consistency of this link vary significantly depending on exposure definitions (static classification vs trajectory), outcome measurement (clinical diagnosis vs screening vs single-item), and the rigor of confounding control.^[11–21]^ A recurrent and critical insight from male-specific analyses is that the trajectory of worsening stress: rather than a static “high” level at a single-point in time: more clearly predicts incident depression or symptom exacerbation.^[20,21]^ This suggests that mental health risks for male workers are not determined solely by membership in a specific risk group (e.g., shift workers) but are amplified when structural stressors: such as job demands, organizational systems, workplace culture, and job insecurity: deteriorate over time.^[11,20,21]^

Regarding occupational stress, cohort evidence demonstrating that Karasek-based high job strain significantly increases the risk of MDEs in men serves as a foundational signal.^[11]^ Furthermore, in Korean repeated-measures panels, within-person changes in depressive symptoms among men were most consistently linked to increases in job insecurity.^[20]^ This supports the hypothesis that, within volatile economic and employment landscapes, the potential for job loss or uncertainty regarding the future acts as a primary trigger or amplifier of male depression.^[22,23]^ Similarly, studies tracking incident depression exclusively in men observed a sharp rise in risk when multidimensional stressors: including job demands, interpersonal conflict, and rigid workplace culture: increased or transitioned from “low to high” or persisted at “high to high” levels.^[21]^ Consequently, risk management strategies should move beyond binary “high/low” stress classifications toward dynamic monitoring systems that detect early warning signs of stress deterioration, coupled with organizational interventions targeting culture and systemic relationships.^[20,21]^

Evidence linking work arrangements (shift and night work) to depression was heterogeneous. While large-scale cohorts identified clear risk elevations,^[12]^ other studies reported directional increases with high uncertainty^[13]^ or associations that attenuated after adjustment in cross-sectional data.^[19]^ These discrepancies imply that the risk is not captured by a simple “exposure yes/no” metric but is modulated by contextual factors such as shift patterns (fixed night vs rotating), frequency, duration, circadian vulnerability, and recovery time.^[12,13,19]^ Specifically, cross-sectional designs face the challenge of reverse causality, where depressed individuals may alter their schedules or exit the labor market,^[19]^ highlighting the necessity for longitudinal exposure measurement.^[11–13,21]^ From a practical standpoint, mitigating depression risk in shift work environments requires a multilayered approach combining ergonomic shift design (e.g., limiting consecutive night shifts), sleep health interventions, and targeted screening for high-risk groups experiencing stress accumulation.^[12,21]^

Findings regarding long working hours were mixed. Cross-sectional data on young workers demonstrated a clear dose-response relationship between longer hours and depression,^[16]^ whereas male-stratified estimates from long-term panels were weaker or inconclusive.^[15]^ This divergence likely stems from: the varying meaning of long hours across age groups and occupations (e.g., voluntary vs forced overtime, rewards, autonomy); the “healthy worker effect” in long-term follow-ups, where men susceptible to depression may systematically exit the workforce or reduce hours^[15]^; and differences in instrument sensitivity (single-item vs validated scales).^[16]^ Furthermore, the relationship may be nonlinear; studies employing IV approaches have identified a U-shaped curve, suggesting that both underemployment and overwork pose risks.^[18]^ Policy interventions should therefore look beyond uniform time limits and assess whether long hours are compounded by high demands or a toxic culture (e.g., norms enforcing overtime), with a focus on preemptive protection for young men and those in high-intensity roles.^[16,18]^

### 5.1. Future research and minimum measurement standards

To improve the clinical equivalence of depression outcomes, future occupational studies should use, at minimum, a validated multi-item measure with a prespecified scoring rule and cutoff. Preferably, self-report instruments (e.g., PHQ-9 or CES-D) should be combined with a validated observer- or clinician-rated instrument (e.g., Hamilton Depression Rating Scale or Montgomery-Åsberg Depression Rating Scale) or a structured diagnostic interview in the full cohort or a validation subsample. Studies should report the instrument version and language, assessment window, cutoff, continuous score, and timing relative to exposure; a single-item depression measure should not be used as the sole outcome.

Also, for occupational stress, a minimum exposure set should combine an established psychosocial framework - such as the Karasek Job Content Questionnaire or KOSS, with domain-level scores - with precise schedule metrics: number of night shifts per month, shift duration, consecutive night shifts, rotation direction and speed, weekly working hours, overtime, intershift rest, and duration in the schedule. Repeated assessments should capture exposure trajectories, and reports should stratify or adjust for age, occupation, employment stability, baseline mental health, sleep, and major socioeconomic confounders. Adopting this core set would permit clinically interpretable cross-study comparisons and more defensible pooling.

This review has several limitations. First, substantial heterogeneity in study design and definitions precluded quantitative meta-analysis, necessitating a qualitative synthesis.^[11–21]^ Second, depression outcomes ranged from clinical diagnoses (MDE, medical records) to screening tools and single items, limiting clinical equivalence; single-item measures, in particular, carry a high-risk of misclassification.^[12,16–18]^ Third, reliance on cross-sectional designs or single-point exposure measurements in several studies restricted the assessment of temporal precedence and exposure changes,^[14,16,19]^ while selection bias and residual confounding remained concerns.^[13,15,19,20]^ Finally, consistent male-stratified effect estimates were not available across all studies, limiting precise comparisons by age, occupation, or employment type.^[12,15,17,20]^ Consequently, these findings warrant cautious interpretation. Fifth, the review was not prospectively registered, and the absence of a public protocol may increase the risk of selective methodological decisions or reporting despite use of a prespecified internal working protocol.

## 6. Conclusion

Collectively, this systematic review supports the potential association between occupational stress, nonstandard work arrangements, and depression in men. Specifically, the deterioration of job demands, job insecurity, and organizational culture appears strongly linked to incident depression. While the effects of shift work and long working hours show heterogeneity across studies, elevated risks have been confirmed in large-scale and male-specific cohorts. Future research must strengthen causal inference through longitudinal designs that account for male-specific subgroups, utilize standardized depression assessments, and rigorously control for confounding. In the workplace, prevention strategies must evolve from static stress assessments to dynamic monitoring of stress trajectories, integrated with organizational improvements in employment stability and workplace culture.

## Acknowledgments

The author gratefully acknowledges Nnyigide Osita Sunday, PhD (Postdoctoral Researcher, Department of Chemical and Biomolecular Engineering, Pusan National University; Rheology Research Institute), and Okpete Uchenna Esther (Doctoral Researcher and Physician-Scientist, Department of Digital Anti-Aging Healthcare, Graduate School, Inje University). They served as independent reviewers for title/abstract and full-text screening, documented exclusion reasons, and participated in resolving eligibility discrepancies. Their contributions were limited to screening and did not meet the criteria for authorship.

## Author contributions

**Conceptualization:** Haewon Byeon.

**Data curation:** Haewon Byeon.

**Formal analysis:** Haewon Byeon.

**Funding acquisition:** Haewon Byeon.

**Investigation:** Haewon Byeon.

**Methodology:** Haewon Byeon.

**Project administration:** Haewon Byeon.

**Resources:** Haewon Byeon.

**Software:** Haewon Byeon.

**Supervision:** Haewon Byeon.

**Validation:** Haewon Byeon.

**Visualization:** Haewon Byeon.

**Writing – original draft:** Haewon Byeon.

**Writing – review & editing:** Haewon Byeon.
